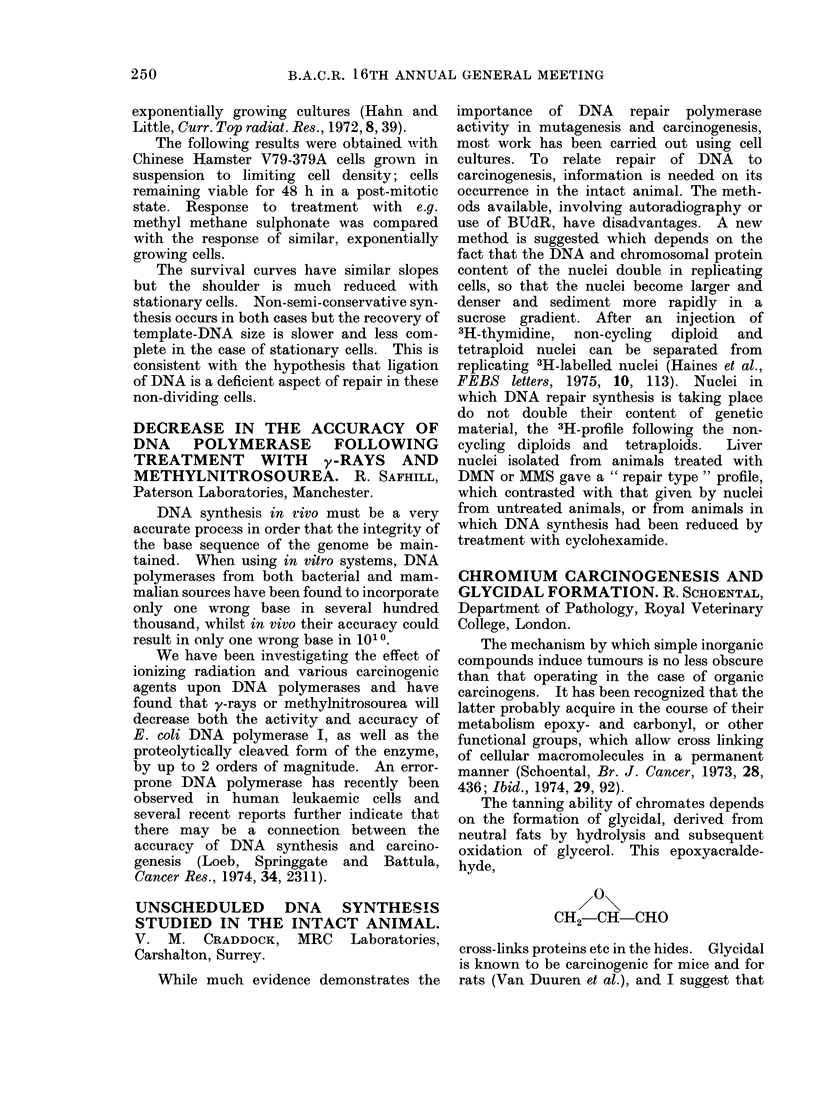# Proceedings: Unscheduled DNA synthesis studied in the intact animal.

**DOI:** 10.1038/bjc.1975.189

**Published:** 1975-08

**Authors:** V. M. Craddock


**UNSCHEDULED DNA SYNTHESIS STUDIED IN THE INTACT ANIMAL.** V. M. CRADDOCK, MRC Laboratories, Carshalton, Surrey.

While much evidence demonstrates the importance of DNA repair polymerase activity in mutagenesis and carcinogenesis, most work has been carried out using cell cultures. To relate repair of DNA to carcinogenesis, information is needed on its occurrence in the intact animal. The methods available, involving autoradiography or use of BUdR, have disadvantages. A new method is suggested which depends on the fact that the DNA and chromosomal protein content of the nuclei double in replicating cells, so that the nuclei become larger and denser and sediment more rapidly in a sucrose gradient. After an injection of $^{3}$H-thymidine, non-cycling diploid and tetraploid nuclei can be separated from replicating $^{3}$H-labelled nuclei (Haines *et al.*, *FEBS letters*, 1975, **10**, 113). Nuclei in which DNA repair synthesis is taking place do not double their content of genetic material, the $^{3}$H-profile following the non-cycling diploids and tetraploids. Liver nuclei isolated from animals treated with DMN or MMS gave a " repair type " profile, which contrasted with that given by nuclei from untreated animals, or from animals in which DNA synthesis had been reduced by treatment with cyclohexamide.